# Nonlinear effects of built environment on metro-feeder bike-sharing as a dynamic green exposure pathway: a dual perspective of connection frequency and modal share

**DOI:** 10.3389/fpubh.2026.1951162

**Published:** 2026-10-27

**Authors:** Lei Pang, Cailin Qiu, Haijun Sun, Xi Ren, Zhong Sun

**Affiliations:** 1 School of Civil Engineering and Architecture, University of Jinan, Jinan, China; 2 School of Architecture and Urban-Rural Planning, Fuzhou University, Fuzhou, China; 3 Department of Urban and Rural Planning, Southwest Jiaotong University, Chengdu, China; 4 School of Architecture, Nanyang Institute of Technology, Nanyang, China

**Keywords:** built environment, connection frequency, green view index, metro-feeder bike-sharing, modal share, nonlinear effects

## Abstract

**Background:**

Bike-sharing integrated with metro transit can be conceptualized as a potential dynamic green exposure pathway linking blue-green infrastructure, active mobility, and public health. The built environment is a key determinant, but existing research focuses on connection frequency and neglects modal share, which better reflects feeder efficiency and competitiveness.

**Objective:**

This study compares built environment effects on metro-feeder bike-sharing connection frequency and modal share and identifies nonlinear thresholds.

**Methods:**

Using Tianjin, China as a case study, we integrated multi-source travel data with geospatial data. The Green View Index (GVI), derived from Baidu Street View via deep learning, served as a station-catchment-level proxy for potential street-level green exposure. Actual cycling trajectories and health outcomes were not measured. A LightGBM-SHAP model quantified variable contributions and uncovered nonlinear associations.

**Results:**

Three findings emerge. First, connection frequency concentrates in the city center, whereas modal share clusters in suburban ring zones, suggesting spatial unevenness in access to bike-sharing as a green feeder mode. Second, drivers differ: connection frequency is mainly explained by governmental (39.8%), commercial (11.5%), and cultural-educational POI density (9.4%), reflecting commuting-oriented mechanisms; modal share is dominated by healthcare POI density (27.0%), distance to city center (15.0%), and bus stop density (10.0%), highlighting accessibility and inter-modal competition. Third, nonlinear thresholds are pervasive: governmental POI density turns positive beyond 30 POIs/km^2^; GVI above 90 is associated with positive feeder cycling; bus stop density beyond 4 stops/km^2^ shifts from complementarity to competition; and floor area ratio has an inverted U-shaped effect on modal share, positive at 0.5–2.1.

**Conclusion:**

This study is the first to quantitatively disentangle the differentiated nonlinear effects of the built environment on bike-sharing connection frequency and modal share from both “volume enhancement” and “efficiency optimization” perspectives. The identified thresholds provide evidence-based planning guidance for optimizing metro-feeder bike-sharing systems and supporting climate-adaptive and more spatially equitable green feeder access. Because GVI is a station-catchment proxy and health outcomes were not measured, the health-equity implications are interpretive rather than causal.

## 1 Introduction

Bike-sharing has rapidly gained widespread adoption in numerous Chinese cities as a green, low-carbon, convenient, and efficient travel mode. It has effectively addressed the “last-mile” challenge of metro access, emerging as a core component of the integrated “metro + active travel” green transport system (1–3). Riding to and from metro stations not only provides commuters with routine opportunities for physical activity—helping to reduce the risk of cardiovascular diseases, obesity, diabetes, and certain cancers, thereby directly promoting active mobility and public health (4, 5)—but also contributes to curbing short-distance motorized trips, lowering transportation-related carbon and air pollutant emissions, and improving urban environmental quality (6, 7). However, whether these potential health and environmental benefits are equitably realized depends not solely on the spatial provision of facilities, but more critically on residents’ actual ability to access and benefit from the active travel environment in their daily routines, namely the spatial accessibility and exposure quality of “green exposure pathways.” Therefore, conceptualizing bike-sharing access to metro stations as a key dynamic green exposure pathway is of great significance for understanding how urban transport systems can leverage infrastructure synergies to achieve both health equity and environmental improvement.

The built environment is widely acknowledged as a key factor influencing bike-sharing usage for metro access (8). In recent years, with the widespread availability of smart card transaction data, bike-sharing GPS trajectories, and multi-source geospatial data, scholars have increasingly leveraged big data to examine the relationship between the built environment and feeder behavior. However, most existing studies have concentrated on the effects of the built environment on connection frequency, namely the intensity of usage (9–11), while largely overlooking its influence on modal share. Connection frequency reflects only the absolute volume of trips and fails to capture the functional efficiency and relative competitiveness of bike-sharing within the feeder system. Within the theoretical framework of public transport resource allocation and mode split, the feeder modal share—defined as the proportion of bike-sharing connection trips to total metro station ridership—serves as a critical indicator for assessing the coordination efficiency of the “metro + active travel” system and the actual capacity of green feeder services. To date, research has not provided differentiated theoretical or spatial planning support from the dual perspective of “volume enhancement” and “efficiency optimization”.

To address this gap, this study aims to answer the following two research questions: (1) What are the spatial differences between metro-feeder bike-sharing connection frequency and modal share? (2) How do the associations between the built environment and connection frequency differ from those between the built environment and modal share? To answer these questions, we first measured built environment indicators across multiple dimensions using multi-source built environment data. Second, we employed bike-sharing GPS data and a multi-ring buffer analysis method to precisely delineate the feeder service catchment areas, and then combined these data with metro smart card records to separately calculate connection frequency and modal share. Finally, we constructed a LightGBM-SHAP model to quantify the contribution of built environment factors to both feeder indicators and to uncover the nonlinear relationships between each factor and the two outcomes. The findings of this study can assist urban planners in optimizing the built environment in a targeted manner, taking threshold effects into account, so as to simultaneously enhance both the connection frequency and modal share of metro-feeder bike-sharing.

In this study, we define a dynamic green exposure pathway as the routine active-travel route through which metro passengers access or egress from stations by bike-sharing, during which they are exposed to street-level greenness and accumulate physical activity. The term “dynamic” emphasizes that exposure occurs during daily movement rather than at a fixed residential location, while “pathway” emphasizes the link between blue-green infrastructure, active mobility, and public health. The Green View Index (GVI) is employed as a proxy for visual green exposure along this pathway. Health equity, in this context, refers to the equitable spatial distribution of opportunities to use bike-sharing as a green feeder mode, and thus to gain routine physical activity and green exposure. This study does not measure health outcomes directly; rather, it examines whether the built environment supports green feeder access evenly across space.

This study takes Tianjin, China, as a case study and designs an analytical framework. Section 2 reviews the relevant literature, covering the evaluation dimensions of built environment elements and their relationships with metro-feeder bike-sharing behavior. Section 3 describes the study area, methods, and datasets. The methods include deep learning-based segmentation and decision tree models. The datasets consist of metro smart card records, bike-sharing GPS trajectories from two operators (Meituan and Qingju), Baidu Street View imagery, and other built environment data. Section 4 presents the empirical results, including the determination of the feeder buffer zone extent, the spatial distribution of connection frequency and modal share, the collinearity test of built environment independent variables, the contribution analysis of built environment factors, and their nonlinear effect characteristics. Finally, Section 5 provides discussion and conclusions, proposes planning recommendations based on the findings, and outlines directions for future research.

## 2 Literature review

### 2.1 Relationship between bike-sharing and metro

Since its emergence in China around 2015, bike-sharing has rapidly become a prominent mode of short-distance urban travel, owing to its dockless flexibility and the convenience of smartphone-based unlocking. The integration of bike-sharing with metro transit constitutes a critical pathway for addressing the “last-mile” problem. In major Chinese cities, over 60% of bike-sharing trip origins and destinations are located within the vicinity of metro stations, indicating that bike-sharing has become a significant feeder mode for metro systems (8, 12).

From the perspective of travel behavior theory, two fundamental relationships exist between bike-sharing and public transport: substitution and integration. Substitution refers to bike-sharing directly replacing trips that would otherwise have been undertaken by public transport, whereas integration denotes bike-sharing serving as an extension of public transport, thereby expanding its service coverage (13). A key objective of urban transport planning is precisely to promote the integrative function of bike-sharing with public transport, so as to substitute car travel and enlarge the service catchment of public transit. Within this integrative relationship, bike-sharing can expand the service catchment of metro stations by three to five times compared with walking, while offering greater environmental benefits and flexibility than bus transfers or car-based access (14).

Identifying the integrated usage relationship between bike-sharing and metro systems requires first addressing the challenge of linking two distinct travel data sources. In bike-sharing systems, rental and return processes are completed entirely through mobile applications and belong to a different data system from metro smart card records, rendering direct linkage impossible. To address this issue, existing studies typically delineate buffer zones of a specified radius around metro station entrances and exits, and consider bike-sharing trips with origins or destinations falling within these buffers as interacting with the metro station. Regarding the determination of buffer radius, Guo et al. (15), drawing on prior experience and questionnaire surveys, identified areas with high and medium concentrations of bike-sharing parking within a 100-meter buffer around metro stations as feeder zones. Cheng et al. (16) further validated the feasibility of this approach. Wu et al. (17), by calculating the distance between bike-sharing parking points and the nearest metro station and observing the probability density distribution of all trips, refined the relationship between bike-sharing and metro stations into three types: connection, competition, and complementation.

### 2.2 Evaluation dimensions of built environment elements

The influence of the built environment on travel behavior remains a longstanding and central topic in transport geography and urban planning. In 1997, Cervero and Kockelman (18) introduced the “3D” evaluation framework, which characterizes the built environment along three dimensions: Diversity, Density, and Design. Subsequently, Ewing and Cervero (19) augmented this framework by incorporating two additional dimensions—Distance to transit and Destination accessibility—thereby establishing the “5D” framework. This framework has since become a foundational theoretical tool for analyzing the relationship between the built environment and travel behavior.

In recent years, with the widespread availability of big data, researchers have quantified the aforementioned dimensions using multi-source geospatial data, including POI data, street view imagery, and road network data (20–22). Numerous studies have sought to introduce new indicators to enrich the “5D” evaluation framework, such as refined land use classification (23), building characteristics (24), and green space metrics (e.g., Green View Index, NDVI) (25, 26). Concurrently, some researchers have employed complex network analysis to investigate the topological features of metro networks (27), including station betweenness centrality and closeness centrality (28). These emerging indicators afford novel perspectives for a more comprehensive assessment of the built environment.

### 2.3 Relationship between built environment and bike-sharing feeder behavior

#### 2.3.1 Research perspective

Previous studies have largely centered on the influence of the built environment on bike-sharing connection frequency, namely usage intensity. For instance, Chen et al. (29) applied a partial least squares regression model in Nanjing and found that built environment attributes—including population density, number of restaurants, and bicycle parking facilities—exerted significant positive effects on metro-feeder bike-sharing ridership. Guo (15) employed a negative binomial regression model in Shenzhen and demonstrated that land use mix, station proximity to the city center, and high metro ridership collectively promoted the integrated use of bike-sharing and metro services. Zhou et al. (9) utilized a multiscale geographically weighted regression (MGWR) model in Beijing and revealed that the effects of built environment factors on the integrated use of bike-sharing and public transport exhibited significant spatial heterogeneity.

Other scholars have investigated the influence of the built environment on bike-sharing transfer distance. For example, Li et al. (30) applied a GWR model in Shanghai to explore its spatial variations, providing empirical evidence to inform the optimization of bike-sharing deployment and scheduling strategies around metro stations. Nevertheless, existing studies have predominantly concentrated on connection frequency and transfer distance, while paying insufficient attention to feeder modal share—an indicator that more directly reflects the functional efficiency and relative competitiveness of the feeder system. Consequently, they fail to fully capture the actual contribution of bike-sharing within the “metro + active travel” system.

#### 2.3.2 Research method

In recent years, a growing body of evidence has indicated that the effects of the built environment on bike-sharing feeder behavior are not spatially homogeneous. Traditional global regression models, such as ordinary least squares, assume that the relationships between variables remain constant across the entire study area—an assumption that often proves untenable in research on the built environment and travel behavior. To capture spatial heterogeneity, researchers have progressively introduced geographically weighted regression (GWR) and its enhanced variant, multiscale geographically weighted regression (MGWR) (31).

Zhou et al. (9) conducted a case study in Beijing and distinguished two feeder scenarios: “bike-sharing to bus” and “bike-sharing to metro”. They found that the MGWR model significantly outperformed the ordinary least squares (OLS) model in terms of both goodness-of-fit (R^2^) and AICc, confirming that the influence scales of different built environment factors vary. For example, factors such as educational and cultural facilities and bicycle lane length exert their effects at larger bandwidths, whereas bus stop accessibility exhibits more localized spatial effects. Li et al. (32) focused on bike-sharing usage around metro stations in Beijing and similarly validated the superiority of MGWR over OLS and traditional GWR models. They further revealed that the density of residential-hotel POIs and arterial road density influence feeder usage at the global scale, while distance to the CBD and population density operate at the regional scale.

In addition to spatial regression models, machine learning methods are garnering increasing scholarly attention. A number of studies have begun to employ machine learning approaches, including gradient boosting decision trees (GBDT) and extreme gradient boosting (XGBoost) (21, 22, 33), and have found that the effects of built environment factors on bike-sharing feeder behavior frequently exhibit nonlinear patterns and threshold effects. For instance, Song et al. (34) conducted a systematic investigation in Shanghai using the XGBoost model combined with the SHAP interpretation method at a 500-meter grid scale. They found that key built environment factors—including floor area ratio, population density, and road network density—all exhibited significant threshold effects. Moreover, the direction and magnitude of these effects varied markedly between different areas, such as Puxi and Pudong, providing evidence for context-specific planning interventions. Zhuang et al. (35) employed the GBDT model and found that street-level Green View Index (GVI) and Sky View Index (SVI) exerted threshold effects on cycling behavior. Specifically, when GVI fell below 0.2 or SVI below 0.1, their positive effects on bike-sharing usage gradually diminished. This suggests that optimizing the built environment does not necessarily yield greater benefits with increased investment; rather, it exhibits a “plateau effect” characterized by diminishing marginal returns.

### 2.4 The limitations of the existing research

Taken together, Sections 2.1–2.3 reveal three interconnected but unresolved issues. First, although Section 2.1 established that bike-sharing functions as an integrative feeder mode and that buffer-based methods are widely used to identify metro-feeder trips, the literature has not moved beyond identifying integration itself to evaluating how efficiently bike-sharing performs relative to other feeder modes. Second, Section 2.2 showed that the 5D framework and its extensions have enriched built environment measurement, yet green exposure has rarely been treated as a dynamic pathway variable linking active mobility to health equity. Third, Section 2.3 demonstrated that while the research perspective has remained centered on connection frequency and methods have advanced from global regression to spatially varying and machine learning models, these advances have not been used to systematically compare the nonlinear effects of the built environment on connection frequency and modal share. These three gaps motivate the dual-perspective framework adopted in this study.

First, there is a conspicuous gap in research perspective. Previous studies have predominantly concentrated on connection frequency, namely usage intensity (36), while paying insufficient attention to feeder modal share, which more directly reflects the functional efficiency of the feeder system. Connection frequency and modal share correspond to two distinct dimensions—“volume enhancement” and “efficiency optimization,” respectively—and the underlying mechanisms driving these two outcomes may be fundamentally different. Nevertheless, no prior study has systematically compared the differential effects of the built environment on these two feeder indicators.

Second, the majority of existing research has addressed the built environment and bike-sharing feeder behavior from the perspective of transport efficiency, with relatively few studies contextualizing this behavior within the framework of green exposure pathways and health equity. As an active travel mode, bike-sharing feeder use is not only an integral component of the transport system, but also constitutes a critical green exposure pathway through which residents gain opportunities for daily physical activity and mitigate the risk of chronic diseases. Residents in different locations experience markedly divergent feeder conditions and green exposure opportunities due to variations in built environment configurations. Yet this issue remains insufficiently examined from a health equity perspective.

Third, although machine learning methods have revealed nonlinear and threshold effects of built environment factors on feeder behavior, these methods have rarely been applied to compare the nonlinear mechanisms underlying connection frequency and modal share. As a result, existing evidence cannot support differentiated planning interventions that simultaneously target “volume enhancement” and “efficiency optimization”.

## 3 Materials and methods

### 3.1 Research area

This study selects Tianjin, one of China’s four municipalities directly under the central government, as the study area. Tianjin encompasses over 1,100 square kilometers and is home to more than 13 million residents. As a former “Kingdom of Bicycles,” Tianjin possesses a robust cycling culture and a well-established user base among its inhabitants. Since 2017, bike-sharing has experienced rapid expansion in Tianjin. By December 2020, the city had inaugurated and placed into operation six metro lines comprising 143 stations, of which 123 were within the service coverage of bike-sharing (Figure 1).

**Figure 1:**
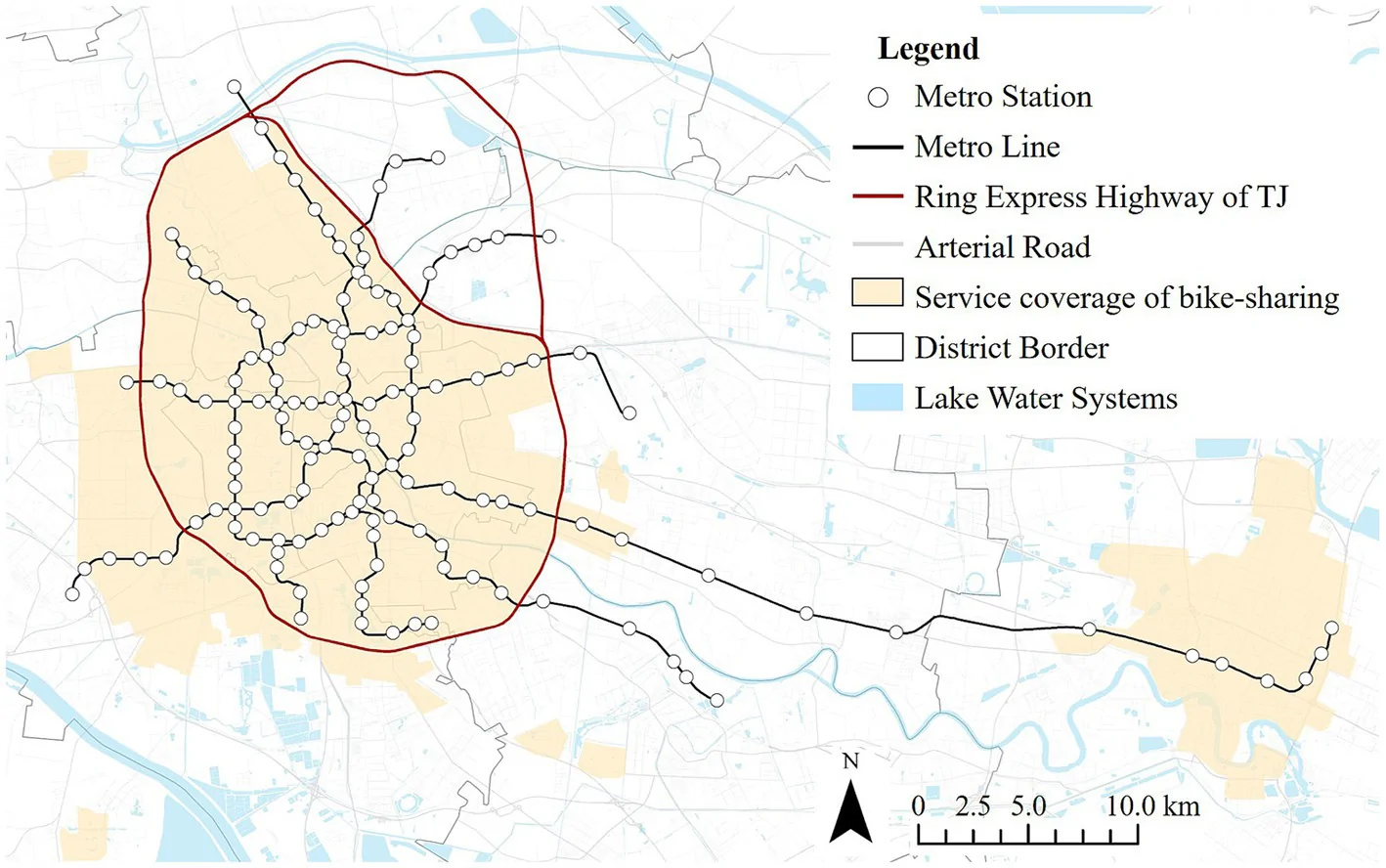
Location of the study area.

In travel behavior research, a radius of 1,500 to 2,000 meters is commonly adopted as the cycling feeder service catchment, based on the 10-min cycling isochrone (15). This study adopts a radius of 1,500 meters as the cycling catchment zone for metro stations. Furthermore, metro stations in the central urban area are relatively densely distributed, and the catchment zones of adjacent stations may overlap. To mitigate this issue, we constructed Thiessen polygons to clip the overlapping areas (37), ultimately obtaining non-overlapping cycling catchment areas for each metro station.

### 3.2 Data and variables

The datasets utilized in this study comprise bike-sharing data from two operators (Meituan and Qingju) in Tianjin, metro smart card data, mobile phone signaling data, and geospatial built environment data, as detailed in Table 1. The bike-sharing data were obtained from the Tianjin Municipal Transportation Commission and include attributes such as positioning time, trip ID, vehicle ID, longitude and latitude coordinates, and lock/unlock status. The dataset covers weekdays from May 22 to 26, 2023 (Monday to Friday). The metro smart card data were sourced from the Transport Planning Research Center of Tianjin Municipal Engineering Institute and include attributes including swipe date, time period, entry station name, exit station name, and origin–destination passenger flow volume. These data cover weekdays from December 14 to 18, 2020 (Monday to Friday). The mobile phone signaling data, provided by China Unicom, were employed to infer residential population distribution.

**Table 1:** Data sources.

Data name	Time	Data source
Tianjin bike-sharing data	May 22–26, 2023	Tianjin Municipal Transportation Commission (raw data provided by Meituan and Qingju operators)
Metro smart card data	December 14–18, 2020	Transport Planning Research Center of Tianjin Municipal Engineering Institute
Tianjin metro station data	December 2020	Baidu Maps platform and Tianjin Rail Transit website (http://www.tjgdjt.com)
Mobile phone signaling data	September–November 2020	China Unicom
Tianjin POIs data	2020	Crawled through the Baidu Maps API (https://lbsyun.baidu.com/)
Tianjin road network data	2020	Collected from Open Street Map (https://www.openstreetmap.org/)
Tianjin bus stop data	2020	Crawled through the Amap API (https://lbs.amap.com)
Building footprint data	2020	Crawled through the Baidu Maps API (https://lbsyun.baidu.com/)
Cycling catchment area data	2020	Collected through the Mapbox Isochrone API (https://docs.mapbox.com/)
Baidu Street View data	September 2021	Crawled through the Baidu Maps API (https://lbsyun.baidu.com/)

The geospatial datasets representing the built environment encompass metro station and network information, POI data, building footprint data, road network data, bus stop data, cycling catchment area data, and Baidu Street View imagery. Specifically, metro station and network data were obtained from the Baidu Maps platform and the Tianjin Rail Transit website. POI data were extracted via the Baidu Maps API, covering 15 POI categories. Building footprint data for Tianjin were also retrieved through the Baidu Maps API, with building floor numbers included. Road network data for Tianjin were collected from Open Street Map, while bus stop data were acquired through the Amap API. Cycling catchment area data for metro stations were obtained via the Mapbox Isochrone API. Baidu Street View imagery was acquired through Python scripts invoking the Baidu Street View API. The imagery data were collected in September 2021, with sampling points placed at 50-meter intervals along road centerlines. At each sampling point, panoramic images were captured from four azimuth angles, resulting in a total of 1.86 million street view panoramic images. For each street-view sampling point, we calculated the Green View Index (GVI) separately for four cardinal directions (north, east, south, and west). For direction *d*, *GVI_d_* is defined as the proportion of green vegetation pixels in the street-view image, expressed as a percentage between 0 and 100%. For each sampling point, we computed the summed *GVI*:

$$GVI_{sum}=GVI_{N}+GVI_{E}+GVI_{S}+GVI_{W}$$

Which ranges from 0 to 400. Station-level GVI is then obtained by averaging *GVI_sum_* across all sampling points within the station catchment.

However, it should be noted that the bike-sharing data were collected in May 2023 and the metro smart card data in December 2020, a gap of approximately 2.5 years. This temporal offset requires explicit justification. Between December 2020 and May 2023, Tianjin’s rail transit network expanded from 6 lines (143 stations, 232 km) to 9 lines (182 stations, 286 km); the 38 new stations were concentrated in suburban and newly developed areas, while the core network serving the central urban districts—the spatial focus of this study—underwent no permanent station closures or relocations. The shared-bike fleet in the central urban districts was capped at 340,000 vehicles in November 2020 and remained subject to this cap through 2023, with the same three licensed operators. December 2020 occurred during a period of normalized epidemic prevention in Tianjin: the municipal measures in force consisted of inbound traveler quarantine, cold-chain inspection, and voluntary travel advisories, with no district-level lockdown or transit service suspension in the central urban districts. These conditions support the use of the two datasets as approximate representations of the same underlying feeder-demand system, although we acknowledge the residual uncertainty discussed in Section 5.3.

### 3.3 Research framework

The technical workflow of this study is structured into three main phases. (1) Using built environment data and mobile phone signaling data, we derived built environment indicators across the “5D + N” dimensions and applied multicollinearity tests to screen the independent variables. (2) By examining the incremental changes in bike-sharing order volumes within multi-ring buffers around station entrances and exits, we ascertained the optimal feeder buffer radius. We then extracted and computed the average daily inbound feeder connection frequency, and derived the average daily inbound passenger flow from metro smart card data to calculate the inbound feeder modal share. (3) We developed a LightGBM-SHAP model to evaluate the contribution magnitude, nonlinear relationships, and threshold effects of built environment factors on bike-sharing connection frequency and modal share. The complete methodological workflow is illustrated in Figure 2.

**Figure 2:**
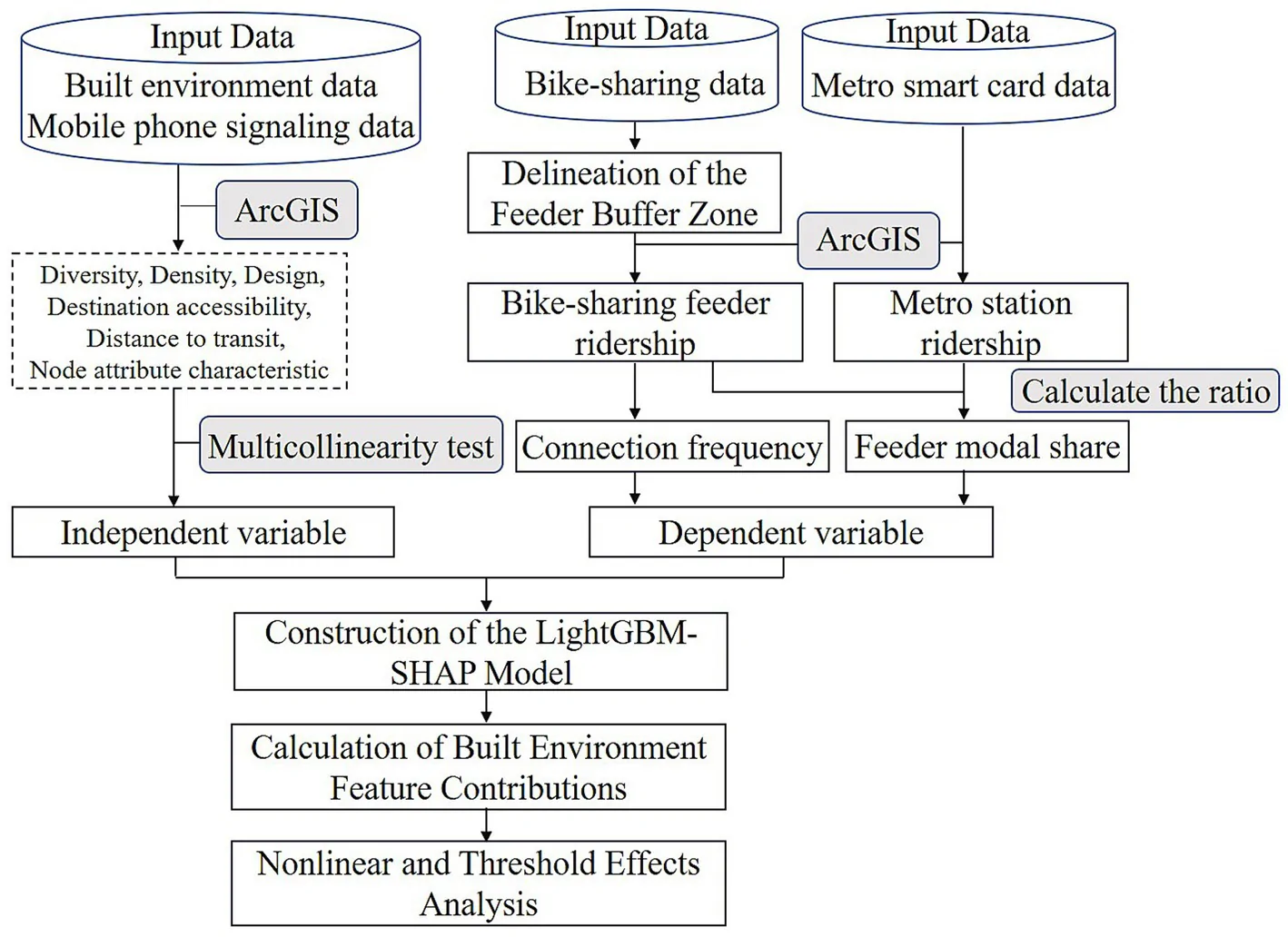
Methodological workflow.

#### 3.3.1 Selection and measurement of built environment indicators

Building upon previous studies (38), this study introduces the node attribute dimension to the “5D” framework, thereby constructing a “5D + N” built environment indicator system, with specific indicators detailed in Table 2. Residential population density was measured using long-term mobile phone signaling data. This was achieved by tracking users’ daily activity trajectories through the positioning function of the signaling data and identifying their residential locations based on activity patterns (39). The Cycling Index represents the 10-min cycling isochrone catchment area originating from the metro station.

**Table 2:** Descriptive statistics of explanatory variables.

Dimension	Indicator	Explanation
Diversity	POI mixing entropy	A POI-based indicator measuring the diversity and balance of urban functions within a spatial unit; higher values reflect greater functional richness and mix
Density	Proportion of residential area(persons/km^2^)	The ratio of nighttime mobile phone signaling counts within the CCA to the area of the CCA
	Density of governmental POIs(POIs/km^2^)	Ratio of governmental POIs to CCA
	Density of commercial POIs(POIs/km^2^)	Ratio of commercial POIs to CCA
	Density of business POIs(POIs/km^2^)	Ratio of business POIs to CCA
	Density of healthcare POIs(POIs/km^2^)	Ratio of healthcare POIs to CCA
	Density of cultural and educational POIs(POIs/km^2^)	Ratio of cultural and educational POIs to CCA
Design	Floor area ratio	Ratio of total gross floor area to CCA
	Road density (m/km^2^)	Ratio of road length to CCA
	Bicycle Riding Index	The area that can be reached within 10 min by cycling starting from the subway station
	Green View Index	An indicator that quantifies the greening level of the urban built environment from the perspective of visual perception
Destination accessibility	Distance from the city center (km)	Yingkoudao Metro Station is taken as the city center
	Bus stops density(units/km^2^)	Ratio of bus stops number to CCA
	Number of entrances and exits(units)	The number of entrances and exits in each metro station
Distance to transit	Average route distance from metro station to bus stops(m)	Average walking route distance from metro station to bus stops
Node attribute characteristic	Is it the starting or ending station	Is it the starting or ending station of the subway line
	Degree	The number of line network edges associated with the nodes
	Network betweenness centrality	A network metric quantifying the extent to which a node or edge lies on shortest paths between other nodes, reflecting its bridging role in controlling information or resource flows
	Network closeness centrality	A network metric defined as the inverse of the sum of shortest path distances from a node to all others; higher values imply faster information propagation and lower reliance on mediators

CCA means the cycling catchment areas of metro stations.

The Green View Index (GVI) was measured using Baidu Street View data, with the PSPNet model employed to segment street view images and calculate the pixel proportion of green vegetation elements. In this study, GVI is positioned as a core indicator of “dynamic exposure-oriented” blue-green infrastructure. Streets characterized by high GVI values typically feature continuous tree canopy coverage (40), which can effectively reduce the equivalent temperature of cycling road surfaces under extreme summer heat conditions, thereby extending the safe time window during which residents can choose bike-sharing as a feeder mode in heatwave events. Moreover, visual exposure to green landscapes has been empirically confirmed to reduce stress levels and facilitate psychological restoration (25, 41).

It should be noted that the GVI is calculated from street-view images sampled along road centerlines within each station’s cycling catchment area, rather than being matched to individual bike-sharing trajectories. Because the available bike-sharing data contain trip origins and destinations but not complete route trajectories, route-specific GVI cannot be reconstructed for every feeder trip. The GVI used here therefore represents the potential street-level green exposure of the feeder environment at the station level, rather than the actual exposure along each individual route. This approach is consistent with previous studies using catchment-level or buffer-level GVI as a proxy for the cycling environment.

Prior to conducting the regression analysis, we performed multicollinearity tests on all built environment independent variables to ensure that the variance inflation factor (VIF) for each variable was below 10.

#### 3.3.2 Determination of feeder service radius based on multi-ring buffer spatial analysis

The optimal feeder service radius for metro stations was ascertained using a multi-ring buffer spatial analysis method. The technical procedure comprises two main steps (Figure 3). (1) Multi-ring buffers were generated around station entrances and exits at intervals of 20 meters, up to a maximum radius of 200 meters. Through spatial overlay analysis, the numbers of bike-sharing unlocking and locking orders within each ring were extracted. (2) An incremental trend analysis method was then applied to examine the evolution patterns of the incremental changes in cycling orders, unlocking, and locking as the buffer radius expanded.

**Figure 3:**
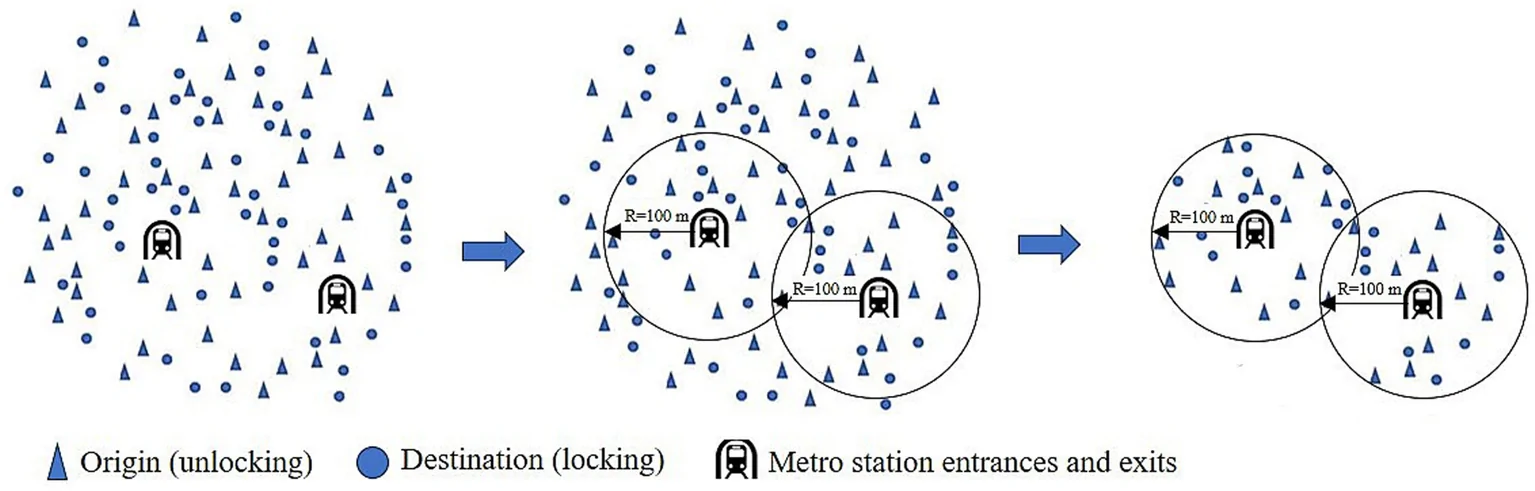
Identification of the integrated travel mode of metro + bike-sharing.

It should be noted that the 100 m buffer approach identifies potential bike-to-metro transfers based on spatial proximity rather than confirmed transfer behavior. A bike-sharing trip ending within 100 m of a station entrance may not necessarily involve metro boarding. However, three considerations support the use of this threshold. First, the 100 m radius was empirically derived from the incremental multi-ring buffer analysis (Figure 4), which showed that bike-sharing order agglomeration stabilized beyond this distance. Second, the 100 m threshold is consistent with previous studies (15, 16) and corresponds to a 1–2 min walking distance, within the psychologically tolerable range for feeder mode choice. Third, because the buffer is tightly drawn around station entrances and operators concentrate bicycle deployment within this zone, the majority of trips ending within 100 m are likely to be feeder-related. Any residual misclassification would introduce random noise rather than systematic bias, and would tend to attenuate rather than inflate the estimated associations.

**Figure 4:**
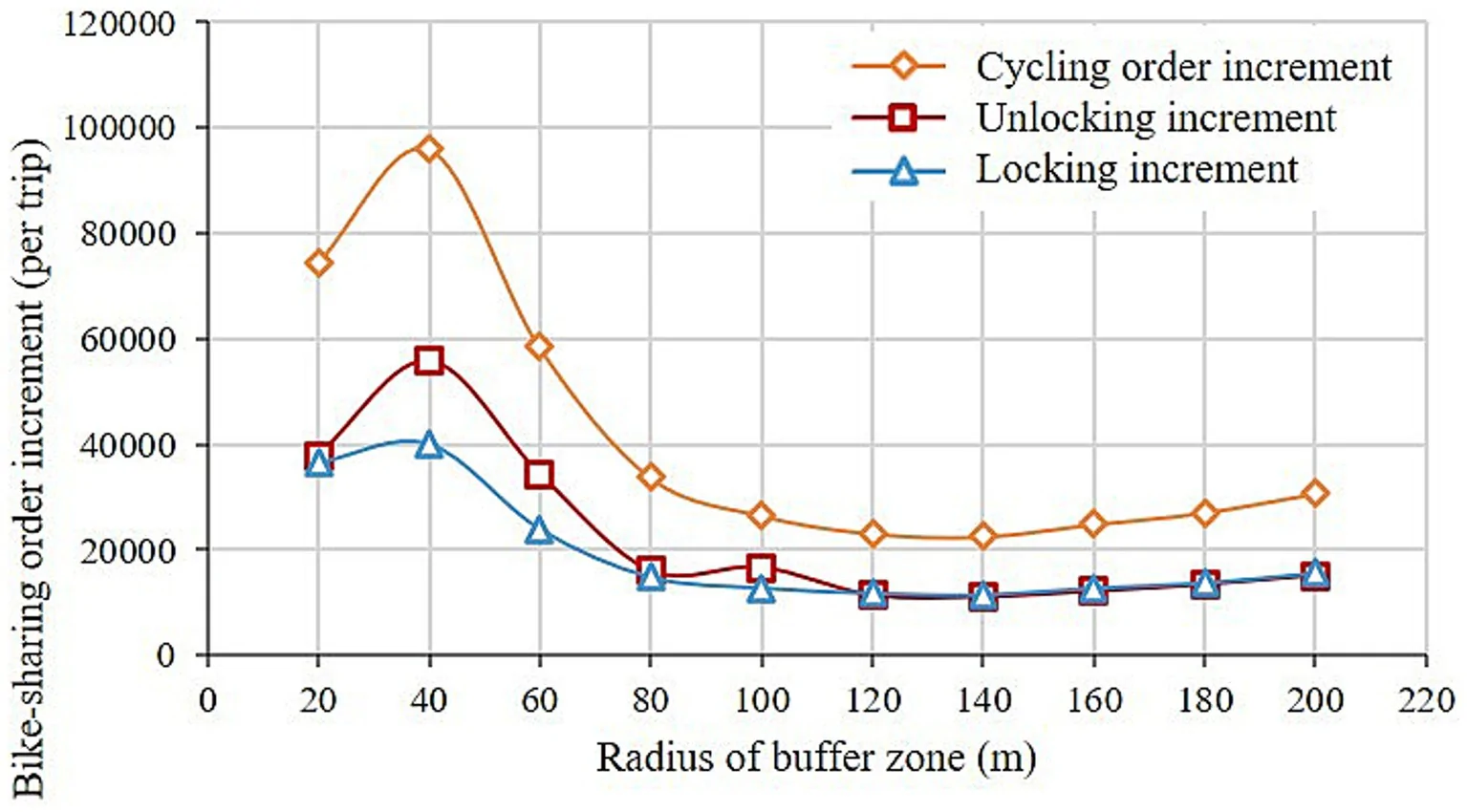
Change in bike-sharing order increment under multi-ring buffers.

#### 3.3.3 LightGBM-SHAP model

This study aims to identify nonlinear threshold effects of built environment factors and to compare these effects between connection frequency and modal share. Spatially varying coefficient models such as GWR and MGWR are well suited for estimating spatially heterogeneous linear relationships, but they cannot directly capture the nonlinear and threshold patterns that are central to our research questions. LightGBM-SHAP was therefore selected because it accommodates high-dimensional nonlinear relationships and provides both global and local interpretations. The SHAP dependence plots reveal how the marginal effect of each variable changes across its value range, and station-level SHAP values allow local decomposition of predictions. Spatial dimensions are further incorporated through distance to city center and other spatially varying predictors, and through the spatial mapping of results.

LightGBM is a GBDT model that is further optimized on the basis of XGBoost. Its core improvement lies in adopting a leaf-wise growth strategy with depth constraints, instead of the traditional level-wise growth method, thereby enhancing training efficiency while maintaining accuracy and mitigating overfitting by limiting tree depth. In the model derivation, the loss function and objective function are defined as follows. Let the number of samples be *N*, the number of decision trees be *M*, *y_i_* denote the true value of the *i*-th sample, and $\hat{y_{i}}$ denote the predicted value.

The loss function $l\left(y_{i},{\hat{y}}_{i}\right)$ is used to measure the error between the true value and the predicted value, and is typically expressed as the mean squared error.

$$l\left(y_{i},{\hat{y}}_{i}\right)={\left(y_{i}-{\hat{y}}_{i}\right)}^{2}$$ (1)

The objective function is defined as follows.

$$objective\left(\theta \right)=\frac{1}{N}\overset{N}{\underset{i=1}{\sum }}l\left(y_{i},{\hat{y}}_{i}\right)+\Omega \left(\theta \right)$$ (2)

Where *θ* denotes the model parameters, and *Ω(θ)* represents the regularization term, which is used to control model complexity.

The objective function is approximated by Taylor expansion and simplification.

$$obj\left(\theta \mathrm{,,}g\mathrm{,,}h\right)=\frac{1}{N}\overset{N}{\underset{i=1}{\sum }}\left[g_{i}f\left(x_{i}\right)+\frac{1}{2}h_{i}f^{2}\left(x_{i}\right)\right]+\Omega \left(\theta \right)$$ (3)

Where *g_i_* and *h_i_* denote the first-order and second-order gradient information of sample *i*, respectively, and *f(x_i_)* denotes the predicted value of the *i* -th sample. The update iteration formula is as follows.

$$f_{m}\left(x_{i}\right)=f_{m-1}\left(x_{i}\right)+\eta h_{m}\left(x_{i}\right)$$ (4)

Where *f_m_(x_i_)* is the predicted value of the model for sample *x_i_* after the *m*-th iteration, *h_m_(x_i_)* is the negative gradient of the *m*-th tree with respect to sample *x_i_*, and *η* is the learning rate.

The final model is as follows.

$$f\left(x\right)=\overset{M}{\underset{m=1}{\sum }}f_{m}\left(x\right)$$ (5)

SHAP (SHapley Additive exPlanations) is a game-theoretic approach for interpreting machine learning models. It quantifies each feature’s contribution to an individual prediction by computing its SHAP value. In contrast to the global feature importance rankings provided by conventional models, SHAP enables both a global assessment of feature influence and a local interpretation of each feature’s contribution to specific sample predictions, thereby effectively transforming “black-box” models into interpretable systems. The SHAP values are derived from the following formula.

$$\emptyset_{i}\left(f\right)=\underset{S\subseteq N\backslash \left\{I\right\}}{\sum }\frac{\left|S\right|!�\left(\left|N\right|-\left|S\right|-1\right)!}{\left|N\right|!}�\left[f\left(S\cup \left\{i\right\}\right)-f\left(S\right)\right]$$ (6)

Where *N* is the set of all features, *S* is a subset of features that does not contain feature *i*, *φ_i_(f)* is the SHAP value of feature *i*, and *f(S)* is the predicted output of the model given the feature subset *S*.

## 4 Results

### 4.1 The spatial distribution characteristics of bike-sharing pick-up and drop-off areas

#### 4.1.1 Connection buffer zone range

Figure 4 illustrates that the incremental increases in cycling orders, unlocking events, and locking events all exhibited a pattern of sharp initial growth followed by a gradual decline, stabilizing after the radius reached 100 m. On this basis, we identified a spatial threshold of 100 m for the feeder buffer zone around metro stations. Within this radius, the agglomeration effect of bike-sharing orders was substantial, providing a scientific foundation for delineating the feeder service catchment. This finding aligns with the conclusions of Guo et al. (15) in Shenzhen, and its validity can be attributed to two primary factors. First, 100 m roughly corresponds to a walking distance of 1 to 2 min, representing the psychologically tolerable range for metro alighting passengers to choose between walking and cycling. Second, in practice, bike-sharing operators tend to prioritize the 100 m area surrounding metro station entrances and exits for bicycle deployment, rendering bike availability within this range significantly higher than in peripheral areas and consequently reinforcing the agglomeration effect. Once the buffer radius exceeded 100 m, the incremental increase in orders leveled off, indicating that bike-sharing usage beyond this range was more likely to serve non-feeder purposes rather than constituting a feeder relationship with the metro.

#### 4.1.2 Frequency of connection and spatial distribution of sharing rate

Figure 5A demonstrates that the inbound connection frequency of bike-sharing on weekdays generally follows a concentric zonal decay pattern from the city center outward, indicating that the metro core area shoulders greater intensity of feeder transport. This spatial pattern closely mirrors the gradient distribution of urban land use intensity, employment density, and population agglomeration. The concentration of high-density commercial and office facilities, together with the substantial residential population in the central urban area, generates considerable “last-mile” connection demand. Moreover, the dense road network and relatively well-developed cycling infrastructure provide a favorable operational environment for bike-sharing feeder services.

**Figure 5:**
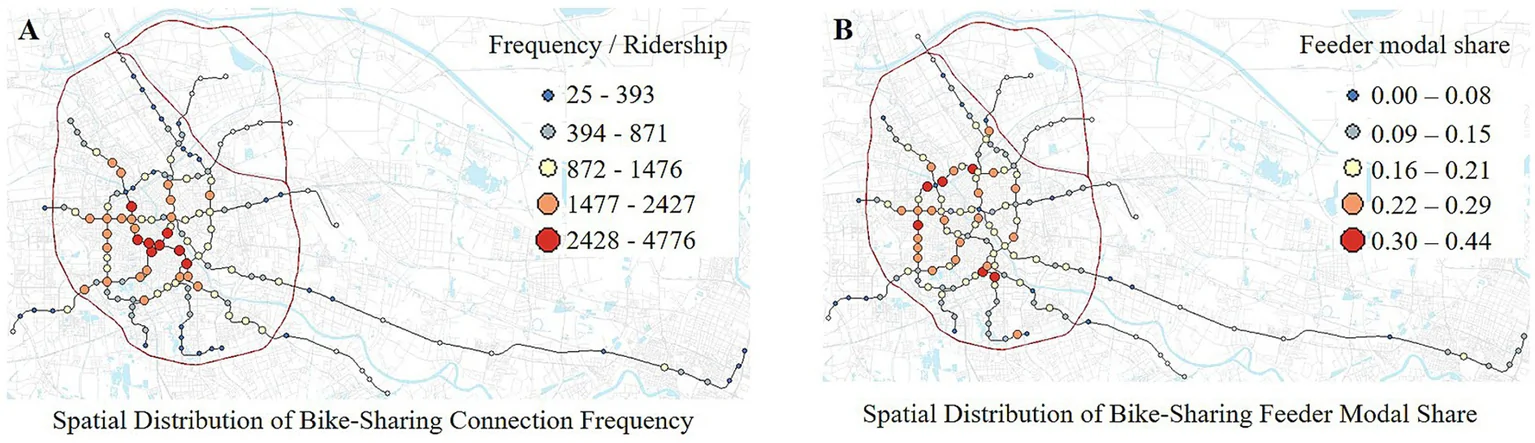
Spatial distribution of bike-sharing connection frequency and feeder modal share on weekdays.

The high-value areas of feeder modal share did not fully coincide with those of connection frequency, but instead clustered predominantly in the suburban ring zones on the periphery of the central urban area (Figure 5B). This spatial divergence reveals that, while bike-sharing is used extensively in the central urban area, its relative competitiveness within the feeder system is more pronounced in specific peripheral zones. In these areas, residential communities tend to be concentrated around stations and feeder distances are moderate, offering an ideal service context for bike-sharing. This pattern is consistent with the feeder dynamics observed at rail transit stations in the Tokyo metropolitan area (42, 43). By contrast, in the central urban area—where stations and facilities are densely distributed—short-distance feeder trips tend to favor walking. In the outer suburbs, however, excessively long feeder distances and inadequate bike supply steer residents toward buses or private motor vehicles for access.

The prevalence of walking substitution for short-distance trips in the central urban area, the feeder efficiency advantage of the suburban ring zones, and the supply deficiency in the outer suburban areas collectively form a comprehensive picture of the spatial differentiation between “usage intensity” and “functional efficiency.” This pattern also reflects the spatial inequity in access to green feeder services.

### 4.2 Descriptive statistics of built environment and optimal model parameter configuration

Table 3 presents the descriptive statistics of built environment factors within the 1,500 m radius catchment area of metro stations. The multicollinearity test results for the built environment independent variables show that all VIF values are below 10, indicating that there is no severe multicollinearity among the independent variables, which meets the fundamental prerequisite for regression modeling (28).

**Table 3:** Descriptive statistics of built environment factors within the cycling catchment area.

Indicator	VIF	Minimum value	Maximum value	Mean value
POI mixing entropy	1.98	0.37	0.96	0.86
Proportion of residential area	2.58	0.08	2.73	0.88
Density of governmental POIs	8.75	0.27	117.87	27.54
Density of commercial POIs	5.05	1.09	408.05	72.48
Density of business POIs	4.71	2.17	421.79	78.00
Density of healthcare POIs	3.83	0.00	84.58	21.60
Density of cultural and educational POIs	6.11	0.00	152.45	29.72
Floor area ratio	5.15	0.04	3.59	1.23
Road density	2.66	2.49	15.61	6.82
Bicycle Riding Index	2.10	0.90	14.41	8.96
Green View Index (0–400 index)	2.59	10.00	282.99	113.74
Distance from the city center	2.96	0.00	47.45	8.46
Bus stops density	3.24	0.00	12.60	4.67
Number of entrances and exits	1.68	1.00	10.00	3.00
Average route distance from metro station to bus stops	1.35	487.79	1,500	985.11
Is it the starting or ending station	1.37	0.00	1.00	0.05
Degree	2.72	1.00	5.00	2.17
Network betweenness centrality	3.93	0.00	0.41	0.09
Network closeness centrality	8.01	0.04	0.13	0.09

GVI_sum_ is the sum of four directional GVI values and is expressed on a 0–400 index scale. It is not a percentage.

The dataset was first split into a training set (70%) and a held-out test set (30%). Hyperparameter tuning was conducted exclusively on the training set using grid search with five-fold cross-validation. The test set was not used during tuning or model selection. After selecting the optimal hyperparameters, the model was retrained on the full training set and evaluated once on the held-out test set.

Table 4 presents the evaluation results of the models for the two dependent variables. The test set R^2^ of the connection frequency model reached 0.72, substantially higher than that of the modal share model (0.40), indicating that the built environment possesses considerably stronger explanatory power for connection frequency than for modal share. This discrepancy stems from the fundamental difference in the nature of the two outcome variables. Connection frequency essentially captures the trip generation process, whose spatial distribution exhibits a direct and stable correspondence with the physical attributes of the built environment. In contrast, modal share reflects the mode choice process, which is more heavily shaped by difficult-to-quantify factors, including individual socioeconomic characteristics, travel habits, and real-time traffic conditions. The influence of the built environment on modal share is exerted indirectly, through its effect on individuals’ subjective perceptions and decision-making preferences, which consequently results in relatively limited explanatory power of the model.

**Table 4:** Optimal parameter configuration and evaluation index results of LightGBM.

Dependent variable	Maximum number of decision trees	Learning rate	Maximum tree depth	Test set evaluation metrics				
				MAE	MAPE	MSE	RMSE	*R* ^2^
Connection frequency	1,000	0.01	3	351.39	57.68	258233.91	508.16	0.72
Connection sharing rate	1,000	0.05	3	0.048	32.53	0.004	0.066	0.40

### 4.3 Contribution analysis

Based on the LightGBM-SHAP model, we computed the mean absolute SHAP values for each variable. The results reveal pronounced disparities in the contribution of individual factors (Figure 6). For connection frequency, the top four contributors—governmental POI density, commercial POI density, cultural and educational POI density, and the Cycling Index—together accounted for a cumulative contribution of 68% (Table 5). This indicates that feeder ridership is predominantly governed by a small set of key factors. Governmental POI density captures the primary driver of commuter connections, as administrative office areas represent the most concentrated origins and destinations for connection demand during morning and evening peak hours. Commercial and cultural-educational POI densities further suggest that daily activities, including shopping, dining, and schooling, are equally important in shaping connection demand.

**Figure 6:**
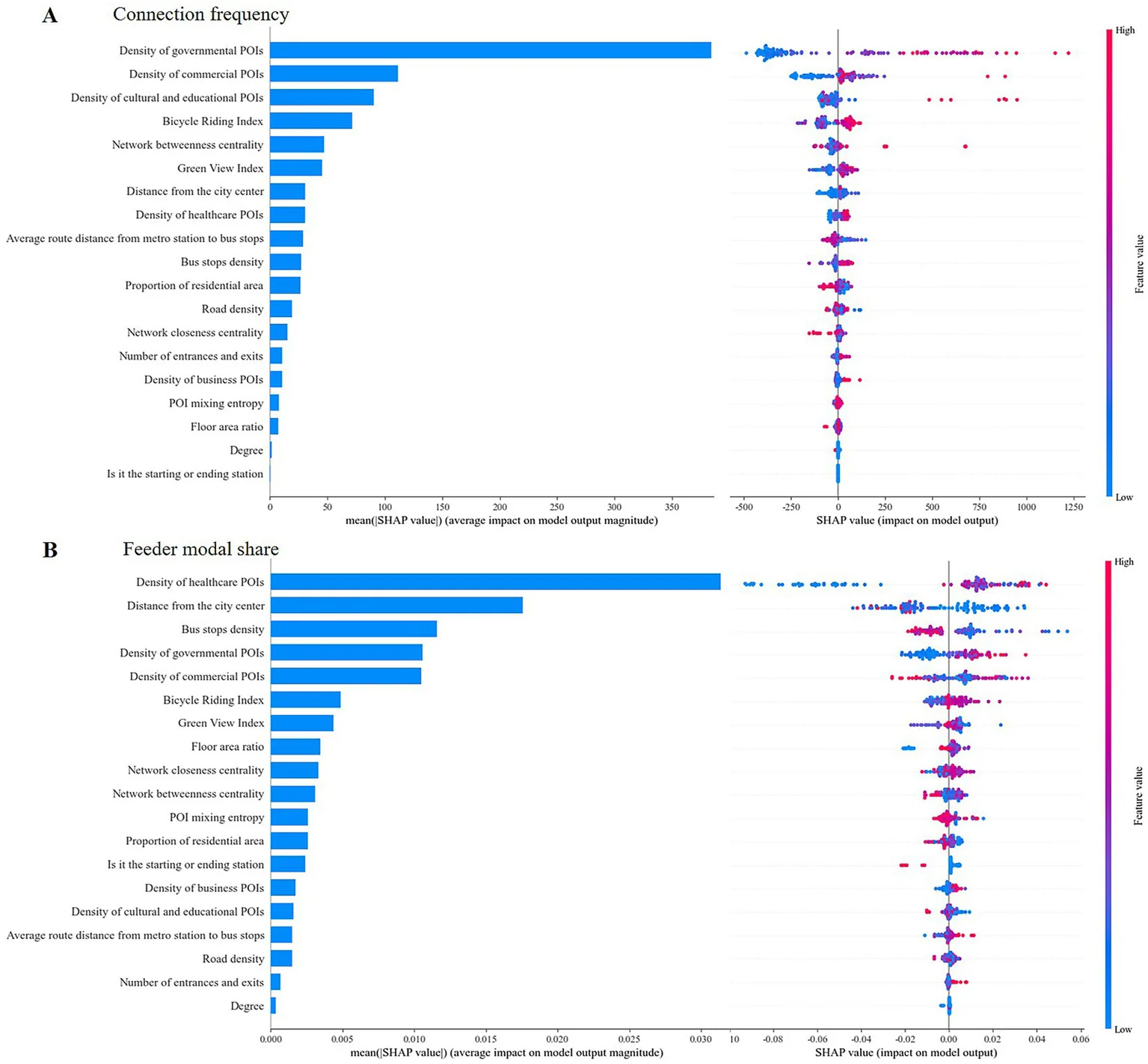
Ranking of built environment factors and distribution of SHAP values.

**Table 5:** Contribution of the built environment.

Dimension	Indicator	Connection frequency		Connection sharing rate	
		Average absolute SHAP value	Proportion /%	Average absolute SHAP value	Proportion /%
Diversity	POI mixing entropy	7.79	0.8	0.0026	2.25
Density	Proportion of residential area(persons/km2)	26.40	2.7	0.0026	2.25
	Density of governmental POIs	384.23	39.8	0.0106	9.17
	Density of commercial POIs	111.33	11.5	0.0105	9.08
	Density of business POIs	10.51	1.0	0.0017	1.47
	Density of healthcare POIs	30.39	3.15	0.0313	27.00
	Density of cultural and educational POIs	90.59	9.4	0.0016	1.38
Design	Floor area ratio	7.33	0.8	0.0034	2.94
	Road density (m/km2)	19.37	2.0	0.0015	1.29
	Bicycle Riding Index	71.42	7.39	0.0049	4.23
	Green View Index	45.25	4.68	0.0044	3.81
Destination accessibility	Distance from the city center	30.78	3.18	0.0176	15.00
	Bus stops density	27.02	2.79	0.0116	10.00
	Number of entrances and exits	10.62	1.10	0.0007	0.60
Distance to transit	Average route distance from metro station to bus stops	28.90	2.99	0.0015	1.30
Node attribute characteristic	Is it the starting or ending station	0.14	0.01	0.0024	2.08
	Degree	1.40	0.15	0.0003	0.26
	Network betweenness centrality	47.01	4.86	0.0031	2.68
	Network closeness centrality	14.96	1.55	0.0033	2.85

For modal share, healthcare POI density emerged as the most influential factor, followed by distance to city center, bus stop density, governmental POI density, and commercial POI density. The top four contributors accounted for 61.5% of the total contribution (Table 5). Healthcare POI density exhibited a positive effect, which may be attributed to the travel demand generated by hospital visitors and healthcare workers, combined with inconvenient parking and limited bus coverage, making bike-sharing a viable complement to metro access. The substantial contribution of distance to city center corroborates the higher modal share observed in suburban areas, underscoring the pivotal role of locational attributes. The Green View Index ranked sixth and seventh in contribution to connection frequency and modal share, respectively. Although not dominant, its contribution was consistent, suggesting that visual greenery exerts a positive influence on cycling, with its effect primarily enhancing the riding experience rather than directly steering travel choices.

### 4.4 Nonlinear analysis

The partial dependence plots generated by the SHAP model reveal the marginal effects of each built environment factor on bike-sharing connection frequency. To facilitate the interpretation of nonlinear patterns, we applied the Locally Weighted Scatterplot Smoothing (LOESS) technique to add fitted curves to the SHAP dependence plots. For descriptive analysis, we selected the top eight contributors, and the results for connection frequency are presented below.

As shown in Figure 7A, governmental POI density exhibited a nonlinear positive correlation with connection frequency. Within the range of 0 to 30 POIs/km^2^, the effect was negative and gradually weakened; beyond 30 POIs/km^2^, the effect turned positive, exhibiting a clear threshold. This shift from negative to positive reflects a transition in the underlying mechanism. Areas with very few governmental facilities offer limited employment opportunities and insufficient commuting demand, leading to naturally low connection frequency. When density reaches 30 POIs/km^2^, job agglomeration generates adequate commuting demand, and the feeder advantage of bike-sharing becomes apparent. Thus, 30 POIs/km^2^ can be regarded as a critical threshold for the activation of commuting demand. Figure 7B demonstrates that commercial POI density also exhibited a nonlinear positive correlation with connection frequency. The effect was negative within the range of 0 to 45 POIs/km^2^ and became positive beyond this threshold, with the intensity first increasing, then decreasing, and subsequently rising again. Specifically, the positive effect strengthened within the 45 to 70 POIs/km^2^ range, then gradually weakened and approached zero, before rising sharply again beyond 240 POIs/km^2^. This complex pattern reflects the superposition of multiple mechanisms. During the low-density phase, commercial facilities are too sparse to constitute stable travel destinations. At the medium-density stage, increased commercial activity generates greater feeder demand. Within the 70 to 240 POIs/km^2^ interval, the effect stabilizes, suggesting that density has become relatively saturated and the marginal benefit of additional facilities diminishes. The pronounced increase beyond 240 POIs/km^2^ is likely attributable to the fact that in highly commercialized areas with dense pedestrian flows and parking constraints, bike-sharing offers greater time efficiency than private cars and taxis, thereby amplifying its competitive advantage.

**Figure 7:**
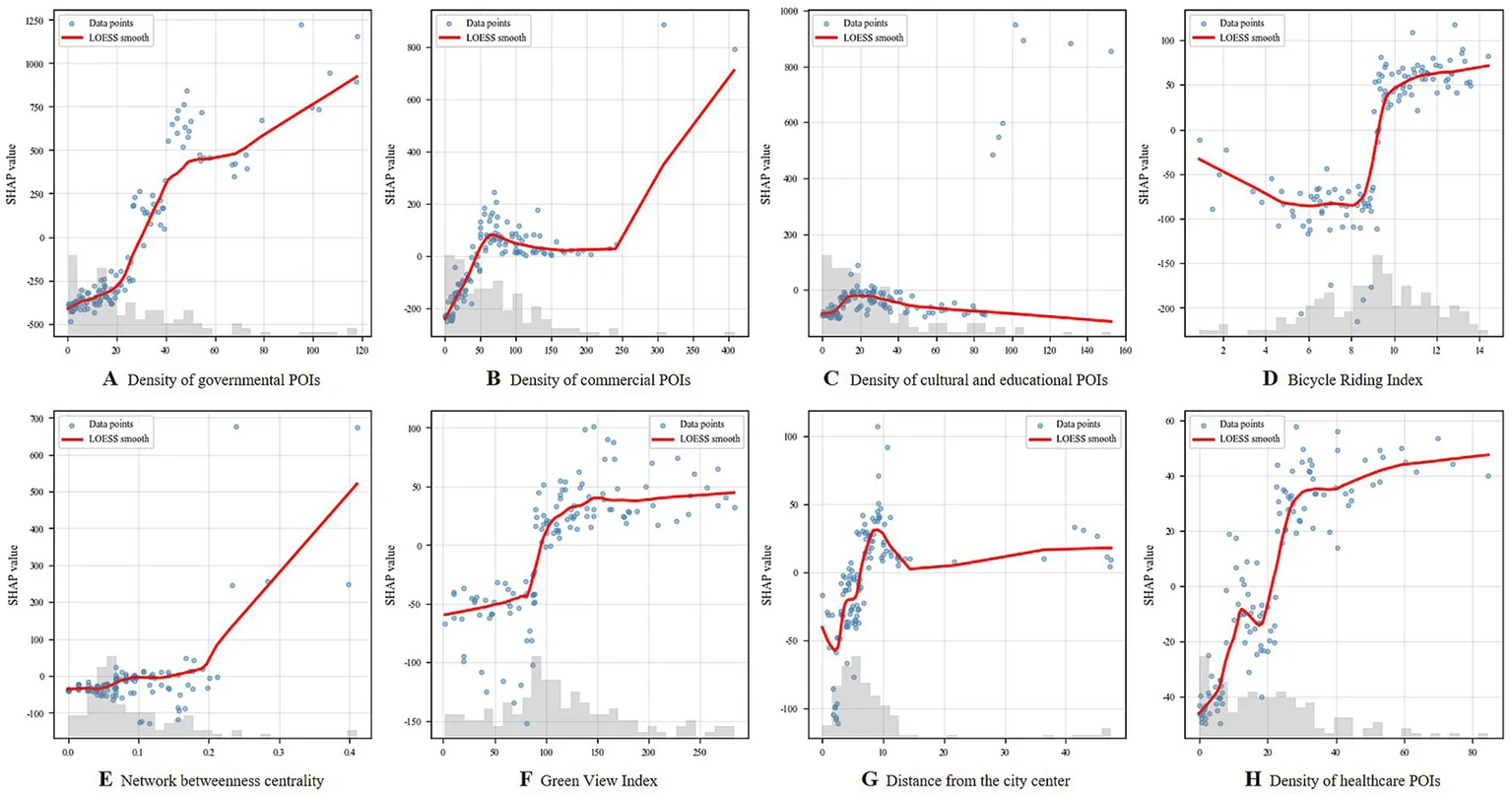
Nonlinear effects of built environment factors on connection frequency.

Figure 7C demonstrates that cultural and educational POI density exhibited a nonlinear relationship with connection frequency. Within the range of 0 to 15 POIs/km^2^, the negative effect gradually weakened; it remained relatively stable between 15 and 25 POIs/km^2^; and beyond 25 POIs/km^2^, the effect became positive and its intensity progressively increased. Cultural and educational facilities primarily serve the school commuting needs of students and staff, and the threshold for their positive effect is relatively low. Figure 7D reveals that the Cycling Index exhibited a nonlinear relationship with connection frequency, characterized by a negative effect followed by a positive one. Within the range of 0 to 9.5, the overall effect was negative, with the negative intensity increasing between 0 and 5, remaining relatively stable between 5 and 8, and then decreasing rapidly between 8 and 9.5. A turning point occurred near 9.5, beyond which the effect became positive and its intensity increased sharply, leveling off around 11. The Cycling Index reflects the cycling catchment area around the station. This turning point suggests that when the catchment area is too small, the feeder advantage of bike-sharing cannot be fully realized, and walking tends to be preferred. Only when the catchment area expands to a sufficient extent can the feeder effect of bike-sharing be fully achieved.

Figure 7E indicates that metro station betweenness centrality exhibited a nonlinear positive correlation with connection frequency. Within the range of 0 to 0.15, the effect was negative with decreasing intensity; beyond 0.15, the effect became positive, and its intensity increased sharply after exceeding 0.19. Stations with high betweenness centrality are typically key nodes where multiple lines intersect or which connect different functional zones, characterized by substantial transfer passenger flows and extensive hinterlands, thereby generating greater feeder demand. The threshold between 0.15 and 0.19 suggests that a station needs to possess sufficient intermediary function for the positive effect to emerge. Figure 7F reveals that the Green View Index (GVI) also exhibited a nonlinear positive correlation with connection frequency. When GVI was below 90, the effect was negative with gradually weakening intensity; beyond 90, the effect became positive and intensified between 90 and 150, then leveled off after exceeding 150. From the perspective of cycling experience, when GVI is too low, the street landscape appears monotonous and the heat island effect is pronounced, which may discourage cycling. When GVI exceeds 90, street trees and green belts provide continuous shade, enhancing riding comfort and thereby increasing connection frequency. Beyond 150, the marginal benefit diminishes, suggesting that moderate greening is sufficient to meet demand and that higher levels do not necessarily yield additional benefits.

Figure 7G reveals that the distance to city center exhibited a nonlinear relationship with connection frequency, characterized by a negative effect followed by a positive effect and then a negative effect again. Within the range of 0 to 7 km, the effect was negative, with the negative intensity initially increasing between 0 and 2 km and then decreasing between 2 and 7 km. A turning point occurred at 7 km, beyond which the effect became positive and reached its maximum intensity at 9 km, then declined again. This three-stage variation corresponds to the concentric zonal characteristics of the city. In the core area within 0 to 7 km, stations are densely distributed and walking access is convenient, limiting the competitive advantage of bike-sharing. In the suburban area between 7 and 9 km, station spacing increases and walking catchment becomes limited, rendering the feeder advantage of bike-sharing more prominent. In the outer suburban area beyond 9 km, feeder distances become excessively long and bike supply is insufficient, leading to a decline in attractiveness. Figure 7H demonstrates that healthcare POI density exhibited a nonlinear positive correlation with connection frequency. Below 22 POIs/km^2^, the effect was negative; beyond this threshold, the effect became positive and increased sharply within the 22 to 30 POIs/km^2^ range, then remained relatively stable. Healthcare facilities are often surrounded by large numbers of older adults individuals and patients who may have concerns about cycling, which could explain the negative effect at the low-density stage. However, when healthcare facilities agglomerate to a certain level, these areas tend to also be high-density residential and mixed-use zones. As a proxy for travel destinations and population activity density, the positive effect of healthcare density gradually outweighs the negative influence associated with the characteristics of healthcare visitors.

The results for feeder modal share are presented below. Figure 8A reveals that healthcare POI density exhibited a nonlinear positive correlation with modal share. Within the range of 0 to 12 POIs/km^2^, the effect was negative; beyond 12 POIs/km^2^, the effect became positive, and its intensity gradually leveled off after exceeding 15 POIs/km^2^. The positive turning point of healthcare POI for modal share was 12 POIs/km^2^, which is lower than the threshold of 22 POIs/km^2^ for connection frequency. This suggests that modal share is more sensitive to the presence of healthcare facilities, and that even in areas with relatively low density, the characteristics of healthcare visitors may exert a discernible influence on mode choice. Figure 8B demonstrates that distance to city center exhibited a nonlinear relationship with modal share, characterized by a positive effect followed by a negative effect. Within the range of 0 to 6 km, the effect was positive, with intensity first increasing and then decreasing, peaking at 5 km. Beyond 6 km, the effect became negative and its intensity continued to increase. Unlike the three-stage pattern (negative–positive–negative) observed for connection frequency, modal share exhibited a simplified pattern of positive followed by negative. The positive interval within 0 to 6 km corroborates the finding that high modal share areas are located in the suburban ring zones, indicating that bike-sharing is more competitive as a feeder mode in suburban areas. The negative effect beyond 6 km may be attributed to the generally lower overall travel intensity in outer suburbs and competition from other feeder modes such as private cars.

**Figure 8:**
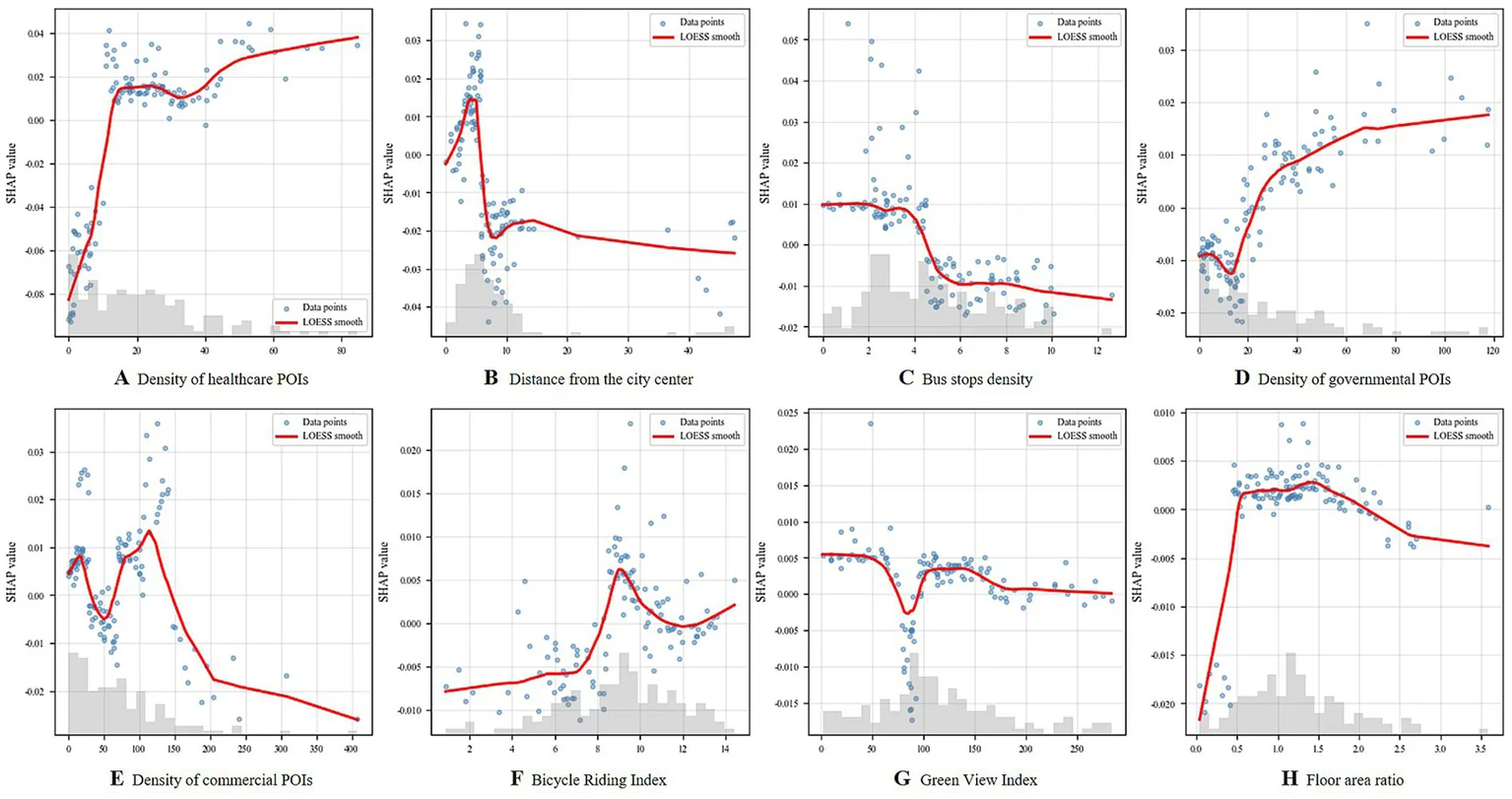
Nonlinear effects of built environment factors on feeder modal share.

Figure 8C reveals that bus stop density exhibited a nonlinear negative correlation with feeder modal share. When the density was below 4 stops/km^2^, the effect was positive; beyond 4 stops/km^2^, the effect became negative and its intensity remained relatively stable. This suggests that when bus facilities are insufficient, bike-sharing primarily plays a supplementary role, with modal share increasing as bus stop density rises. However, once density exceeds a certain threshold, the competitive dynamic between the two modes intensifies, leading to a decline in modal share. This transition from complementarity to competition underscores the complexity of the relationship between buses and bike-sharing. Figure 8D demonstrates that governmental POI density exhibited a nonlinear relationship with feeder modal share, characterized by a negative effect followed by a positive effect. Within the range of 0 to 22 POIs/km^2^, the effect was negative; beyond 22 POIs/km^2^, the effect became positive and increased sharply within the 22 to 30 POIs/km^2^ range, then remained stable. The turning point for modal share was 22 POIs/km^2^, which is lower than the threshold of 30 POIs/km^2^ for connection frequency. This suggests that modal share is more sensitive to the agglomeration of governmental facilities, possibly because commuting modes in administrative office areas are relatively homogeneous, and when employment is highly concentrated, bike-sharing is more likely to achieve scale effects in modal share.

Figure 8E reveals that commercial POI density exhibited a nonlinear relationship with feeder modal share. Within the range of 0 to 30 POIs/km^2^, the effect was positive; it became negative between 30 and 70 POIs/km^2^; then turned positive again between 70 and 150 POIs/km^2^, peaking around 120 POIs/km^2^; and became negative once more with increasing intensity beyond 150 POIs/km^2^. This multi-stage variation reflects the dominance of distinct mechanisms across density intervals. At the low-density stage, commercial activity promotes modal share. At the medium-density stage, improved parking convenience diverts cycling demand. At the high-density stage, extreme congestion reasserts the time advantage of bike-sharing. However, when density becomes excessively high, crowded pedestrian flows and constrained cycling space introduce negative effects. Figure 8F demonstrates that the Cycling Index exhibited a nonlinear relationship with feeder modal share, characterized by a negative effect followed by a positive effect. Within the range of 0 to 8, the effect was negative with intensity gradually weakening; beyond 8, the effect became positive, with intensity first increasing and then decreasing, peaking at approximately 9. The turning point of the Cycling Index for modal share was 8, which is lower than the threshold of 9.5 for connection frequency. This indicates that modal share is more sensitive to the expansion of cycling catchment areas, and that even a moderate increase in catchment size can exert a significant influence on mode choice.

Figure 8G reveals that the Green View Index (GVI) exhibited a nonlinear relationship with feeder modal share, characterized by a positive effect followed by a negative effect and then a positive effect again. Within the range of 0 to 75, the effect was positive but with decreasing intensity; it became negative between 75 and 90; and beyond 90, it turned positive again, with intensity first increasing and then decreasing. Modal share reflects individuals’ trade-off decisions among different feeder modes, and cycling experience represents only one of multiple considerations. The marginal effects of visual environmental changes are moderated by other factors such as weather and trip purpose, which may account for the fluctuating direction of GVI’s influence across different intervals. Figure 8H demonstrates that floor area ratio (FAR) exhibited a nonlinear relationship with feeder modal share, characterized by a negative effect followed by a positive effect and then a negative effect again. The effect was negative within the range of 0 to 0.5; it became positive between 0.5 and 2.1; and turned negative again beyond 2.1. FAR reflects the development intensity of the station area, and its relationship with modal share follows an inverted U-shaped pattern. This indicates that moderate development intensity is conducive to enhancing modal share, as it generates sufficient travel demand to support bike-sharing usage without triggering excessive competition from walking and other feeder modes due to overdevelopment. Both insufficient and excessive development intensity are detrimental to bike-sharing’s share among feeder modes. This finding provides empirical support for the concept that development intensity should be maintained within an appropriate range in TOD planning.

The above analysis collectively demonstrates two important regularities concerning the nonlinear effects of built environment factors on connection frequency and modal share. First, threshold effects are generally pervasive: the influence of most built environment factors on feeder behavior is not monotonically increasing or decreasing, but rather exhibits clear turning points at which the direction or intensity of the effect changes substantially. Second, the same factor frequently exhibits divergent effect patterns on connection frequency and modal share. This finding indicates that the mechanisms underlying trip generation and mode choice are fundamentally distinct in nature, and further suggests that insights gained from enhancing connection frequency cannot be directly generalized to improving modal share.

## 5 Discussions

By integrating multi-source data with the LightGBM-SHAP model, this study systematically reveals, for the first time from the dual perspectives of “volume enhancement” (connection frequency) and “efficiency optimization” (modal share), the differentiated effects and threshold effects of the built environment on bike-sharing feeder behavior. The subsequent discussion elaborates on spatial patterns, contribution characteristics, nonlinear mechanisms, the implications of the Green View Index, and corresponding policy recommendations.

### 5.1 Major findings

This study measures built environment associations with feeder behavior, not actual route-level green exposure or health outcomes. References to green exposure and health equity should therefore be read as planning-oriented interpretations of potential exposure and access equity. This study reveals that high-value areas of bike-sharing connection frequency are concentrated in the city center, whereas high-value areas of modal share are clustered in the suburban ring zones. This spatial “mismatch” profoundly reflects the distinction between the mechanisms of trip generation and mode choice. Connection frequency is driven by high-density activities in the city center and essentially constitutes a direct manifestation of the spatial agglomeration of travel demand. In contrast, modal share depends on the relative competitiveness of bike-sharing against other feeder modes such as walking and buses, reflecting trade-offs at the mode choice level (13, 16). This spatial mismatch raises a potential planning-equity concern regarding access to green feeder environments. Although central-area residents generate higher aggregate cycling volumes, their short-distance feeder trips may often be substituted by walking. Suburban residents, who rely more heavily on bike-sharing, tend to have lower station-catchment GVI. Because actual routes and health outcomes were not measured, these patterns should be interpreted as uneven potential access to green feeder environments rather than as demonstrated health inequities.

Notwithstanding the substantial cycling volume observed in the central urban area, the pronounced time advantage of walking for short-distance feeder trips considerably restricts the modal share of bike-sharing. In the suburban ring zones, where station spacing is moderate and bus coverage is relatively sparse, bike-sharing exhibits greater irreplaceability as a feeder mode. This observation aligns with the comparative study by Lin et al. (44) across Beijing, Taipei, and Tokyo, which concluded that the competitiveness of bike-sharing as a feeder mode varies systematically across different locational contexts, and that direct transfer of policy experience from one city to another may lead to suboptimal outcomes. Guo (15) also demonstrated in Shenzhen that land use types and locational attributes surrounding metro stations exert a significant moderating influence on feeder mode choice behavior.

This finding implies that previous studies, which predominantly employed connection frequency as the sole dependent variable (9, 29), implicitly operated under the assumption that higher cycling volumes equate to superior feeder performance. Such an approach may overestimate the marginal benefits of built environment optimization in the central urban area. The incorporation of modal share as an evaluation metric shifts the analytical focus from “quantitative expansion” to “qualitative optimization,” thereby offering a more comprehensive diagnostic instrument for targeted policy formulation.

The contribution analysis based on the LightGBM-SHAP model reveals that the factor structures shaping connection frequency and modal share are substantially divergent. The top four contributors to connection frequency—governmental POI density, commercial POI density, cultural and educational POI density, and the Cycling Index—are all “commuting and daily activity-oriented” factors, with a cumulative contribution of 68%. This finding is consistent with existing evidence that commuting constitutes the predominant purpose of bike-sharing feeder use (11, 16). By contrast, the top four contributors to modal share—healthcare POI density, distance to city center, bus stop density, and governmental POI density—exhibit characteristics of “accessibility and inter-modal competition-driven” factors. Healthcare POI density ranked foremost, with a contribution of 27.00% in the modal share model. This result reveals a mechanism that has been largely overlooked in prior research: travel associated with healthcare facilities involves older adults and patients, whose feeder mode choice patterns differ fundamentally from those of regular commuters, and who demonstrate heightened sensitivity to cycling safety and comfort (45). The contribution of distance to city center to modal share (15.00%) was substantially greater than its contribution to connection frequency (3.18%), further confirming that locational conditions exert a stronger influence on the efficiency of mode choice than on aggregate travel volume. This corroborates the finding of Zhou et al. (9) in Beijing that the effect of distance to the CBD on the integrated use of bike-sharing and public transport exhibits significant spatial heterogeneity.

This study identifies specific threshold values for multiple built environment variables, furnishing quantitative evidence for fine-grained planning interventions. When governmental POI density falls below 30 POIs/km^2^, employment agglomeration remains insufficient and feeder demand fails to achieve scale effects; beyond this threshold, the rigid demand for commuter connections makes a substantial contribution to connection frequency. This threshold echoes the observation of Song and Diao (34) in Shanghai that the marginal contribution of employment density to bike-sharing usage diminishes beyond a certain level, suggesting the existence of an “effective density range” for employment agglomeration. Commercial POI density below 45 POIs/km^2^ constitutes a negative-effect interval, with 45 to 240 POIs/km^2^ representing a stage of diminishing marginal returns; beyond 240 POIs/km^2^, parking constraints in highly commercialized areas render the time advantage of bike-sharing more salient. When the Green View Index (GVI) falls below 90, the cycling environment is characterized by monotony and a pronounced heat island effect; above 90, continuous shading enhances riding comfort and elevates connection frequency, though marginal benefits diminish after exceeding 150. This threshold aligns in direction with the finding of Zhuang et al. (35) in Shenzhen that the promotional effect of GVI on cycling weakens when GVI drops below 0.2, thereby offering a quantifiable design benchmark for street greening initiatives. It also corroborates the argument of Lu et al. (1) in Hong Kong that GVI more accurately reflects cyclists’ actual green exposure than NDVI. When bus stop density falls below 4 stops/km^2^, bike-sharing assumes a supplementary role; beyond this threshold, the two modes transition to a competitive relationship. This threshold is consistent with the finding of Zhao and Li (46) in Beijing that higher bus stop density correlates with a lower probability of choosing bike-sharing as a feeder mode, indicating that the bus-bike-sharing relationship evolves from complementarity to competition.

This study positions the Green View Index (GVI) as a core indicator of “dynamic exposure-oriented” blue-green infrastructure and establishes that it contributes stably to both connection frequency (4.68%) and modal share (3.81%). Previous investigations have largely concentrated on the effect of GVI on aggregate cycling volume (1, 2), with relatively limited attention to its role in shaping the relative competitiveness of mode choice. Our findings indicate that the influence of GVI is not a simple linear facilitation, but is instead moderated by multiple trip contexts. The SHAP dependence plot for modal share exhibits a “positive–negative–positive” fluctuation pattern, suggesting that the marginal effects of visual environmental changes are conditioned by factors such as weather and trip purpose. From a planning-equity standpoint, GVI is relevant to the spatial distribution of potential green feeder exposure under heatwave conditions. Streets with elevated GVI may help reduce surface temperatures and potentially extend the safe cycling window; however, this study does not measure thermal exposure or health effects. This interpretation is consistent with prior evidence that green exposure relates to physical activity and spatial inequality, but it does not directly test that relationship. In climate-adaptive planning, priority could be given to enhancing street-level GVI in low-income communities and suburban stations, so as to reduce spatial unevenness in potential green feeder exposure.

Although GVI ranked sixth and seventh in SHAP contribution, its effect was stable and consistently positive, and it exhibited clear nonlinear thresholds. GVI is used here as a proxy for street-level greenery that may relate to experiential dimensions such as thermal comfort, safety, and visual restoration; these dimensions were not directly measured. In climate-adaptive planning, shaded cycling corridors are particularly important under heatwave conditions, as they can extend the safe time window for feeder trips. Its policy value therefore lies less in its aggregate explanatory power than in its actionable threshold and its relevance to green exposure equity.

### 5.2 Policy implications

First, differentiated TOD guidance informed by thresholds. Floor area ratio (FAR) exerts a positive influence on modal share within the range of 0.5 to 2.1 and becomes negative beyond 2.1, indicating that planning strategies should transition from “pursuing high density” to “targeting moderate density.” This finding offers empirical validation for the principle that development intensity should be contained within an appropriate range in TOD planning (34).

Second, ring-specific adaptation of feeder services. Drawing on the three-stage influence pattern of distance to city center, we advocate that the central urban area prioritize fine-grained enhancements to the cycling environment, including elevating GVI and improving cycling path continuity. In the suburban ring zones (5–9 km), adequate bicycle deployment and parking facility provision should be ensured to fully capitalize on the feeder competitiveness of bike-sharing. In the outer suburban areas, it is advisable to strengthen the integration of employment and residential functions or to implement coordinated bus-bike-sharing scheduling (16).

Third, collaborative governance between buses and bike-sharing. In areas where bus stop density surpasses 4 stops/km^2^, direct competition should be mitigated through differentiated service positioning—for instance, with bike-sharing focusing on serving bus coverage gaps and buses accommodating longer-distance feeder trips. In areas where density falls below 4 stops/km^2^, bike-sharing should be deployed to supplement insufficient bus service coverage.

Fourth, use GVI as a planning proxy for heat-adaptive cycling environments. We recommend considering a target of GVI ≥ 90 for major feeder roads within 800 m of metro stations, with priority in low-GVI suburban and low-income areas. This target is derived from modeled associations and a station-catchment proxy; it should be treated as a planning hypothesis for improving potential green exposure, not as a verified health intervention. Its purpose is to reduce spatial unevenness in potential green feeder exposure.

Fifth, operational scheduling aligned with thresholds. Bike-sharing operators should intensify bicycle deployment at stations where the built environment approaches “effective thresholds,” and transition to inventory management and dynamic scheduling at stations that have already exceeded these thresholds. Drawing on the method proposed by Lv et al. (11) for predicting bike-sharing trips based on metro station passenger flow characteristics, vehicle scheduling strategies can be optimized by incorporating the threshold attributes of the built environment surrounding each station.

### 5.3 Limitations

The present study is subject to several limitations. First, owing to the unavailability of metro smart card data for non-working days, we were unable to examine feeder passenger flows at each station on weekends and holidays, despite the possibility that feeder behavior patterns during these periods may differ systematically from those observed on weekdays (26). Second, the modal share indicator combines bike-sharing feeder volume (May 2023) with metro ridership (December 2020), introducing two potential biases. The pandemic-period denominator may be understated: Tianjin’s highest single-day metro ridership in 2020 (1.8068 million, January 10) exceeded the December 24, 2020 Figure (1.5616 million) by about 13.6%, suggesting residual suppression of roughly 10–15% in late 2020, which would inflate the modal share by a similar order. The seasonal mismatch further elevates the numerator, since May is near the cycling peak in northern China while December has higher metro use and lower cycling. Together, these biases suggest that the reported modal share should be interpreted as an upper-bound estimate under normal, seasonally matched conditions. Because this scalar bias mainly affects the overall level rather than the cross-station distribution, the spatial patterns of feeder demand remain the primary empirical contribution. Future work with temporally matched data would help refine the point estimate. Third, this study does not provide a quantitative health equity analysis. The concepts of dynamic green exposure pathway and health equity are employed as an analytical framework to interpret the spatial patterns of feeder behavior, rather than as measured health outcomes. The empirical contribution is to identify built environment thresholds that can inform subsequent health equity assessment. Future research could link these thresholds to individual-level health data, such as physical activity levels or heat exposure, to quantify the health equity implications of metro-feeder bike-sharing. Fourth, the GVI was calculated at the catchment level rather than matched to actual bike-sharing travel routes. Because complete trajectory data were unavailable, we could not compute route-specific green exposure. In addition, street greenery benefits both cycling and walking; this study focuses on bike-sharing feeder behavior and does not compare the green exposure of cyclists and pedestrians. Future research could integrate GPS trajectories and walking data to quantify actual route-level exposure and to compare health equity implications across active travel modes. Accordingly, all references to green exposure and health equity in this paper should be understood as referring to potential station-catchment exposure and equity in potential access, rather than to realized route-level exposure or measured health outcomes.

Future research could pursue the following avenues: (1) incorporating the temporal dimension into the modeling framework to analyze seasonal variations in the effects of the built environment on feeder behavior; (2) integrating questionnaire survey data to examine the moderating roles of individual socioeconomic attributes in shaping perceptions of the built environment and feeder mode choice; and (3) extending the analysis to multi-city comparative studies to assess the transferability of the threshold effects identified in the present study.

## 6 Conclusion

This study takes Tianjin as a case study, integrates bike-sharing GPS trajectories, metro smart card data, mobile phone signaling data, and multi-source geospatial data, introduces the Green View Index derived from Baidu Street View imagery, and constructs a LightGBM-SHAP model. From the dual perspectives of connection frequency and modal share, the study systematically elucidates the differentiated nonlinear effects and threshold effects of the built environment on bike-sharing feeder behavior.

At the theoretical level, this study embeds bike-sharing feeder behavior within the analytical framework of “dynamic green exposure pathways,” suggesting that the built environment may indirectly shape opportunities for physical activity and low-carbon travel by influencing feeder mode choice. Actual physical activity, route-level exposure, and health outcomes were not measured. Whereas previous studies have largely confined themselves to one-dimensional analyses of connection frequency or have presupposed linear relationships, this study represents the first systematic comparison of the nonlinear differences between the two indicators, thereby remedying the deficiency inherent in evaluating feeder system performance through a single metric. It furnishes a dual-dimensional diagnostic instrument for the refined assessment of the integrated “metro-active travel” transport system.

At the empirical level, this study yields three principal findings. First, connection frequency and modal share exhibit a spatial misalignment: high-frequency areas are concentrated in the city center, whereas high-modal-share areas are clustered in the suburban ring zones, reflecting regional differentiation between “usage intensity” and “functional efficiency,” as well as potential spatial unevenness in access to green feeder services. Second, the factor structures influencing the two indicators diverge considerably. Connection frequency is predominantly driven by governmental POI density (39.8%), commercial POI density (11.5%), and cultural and educational POI density (9.4%), indicating a commuting and daily activity orientation. In contrast, modal share is primarily shaped by healthcare POI density (27.0%), distance to city center (15.0%), and bus stop density (10.0%), underscoring mechanisms of accessibility and inter-modal competition. The pronounced contribution of healthcare POI density suggests that healthcare-seeking populations exhibit heightened sensitivity to cycling safety and comfort, and that their green exposure opportunities are strongly contingent upon the surrounding built environment, carrying clear implications for health equity. Third, all built environment factors exhibit nonlinear thresholds. For instance, governmental POI density shifts from negative to positive beyond 30 POIs/km^2^; the promotional effect of the Green View Index emerges above 90; bus stop density transitions the relationship with bike-sharing from complementary to competitive beyond 4 stops/km^2^; and floor area ratio manifests an inverted U-shaped effect on modal share, with a positive range between 0.5 and 2.1. These thresholds furnish quantitative evidence to inform fine-grained planning, but their health-equity implications require future linkage to route-level exposure and individual health data.

At the practical level, the Green View Index (GVI), as a station-catchment proxy for potential green exposure, offers a pedestrian-scale indicator for heat-adaptive planning. Streets with high GVI (≥90) may help form shaded corridors, potentially reduce summer cycling surface temperatures, and potentially extend the safe cycling window during heatwaves. We advocate considering a GVI target above 90 for major feeder roads within an 800-meter radius around metro stations as a planning target based on a potential-exposure proxy, not as a guarantee of health benefits. Concurrently, planners should implement differentiated interventions predicated on the identified thresholds: capitalize on the optimal window for TOD development within the FAR range of 0.5 to 2.1; foster differentiated coordination between buses and bike-sharing in areas where bus stop density exceeds 4 stops/km^2^; and ensure that bicycle deployment aligns with demand in the suburban ring zones, so as to fully harness the relative competitiveness of bike-sharing. Therefore, the health-equity implications of this study are planning-oriented and hypothesis-generating; they should not be interpreted as measured health impacts.

## Data Availability

The raw data supporting the conclusions of this article will be made available by the authors, without undue reservation.
